# Behavioral Pain Scale and Critical Care Pain Observation Tool for pain evaluation in orotracheally tubed critical patients. A systematic review of the literature

**DOI:** 10.5935/0103-507X.20190070

**Published:** 2019

**Authors:** Ana Rita Pais de Queiróz Pinheiro, Rita Margarida Dourado Marques

**Affiliations:** 1 Hospital da Luz - Lisboa, Portugal.; 2 Escola Superior de Saúde, Cruz Vermelha Portuguesa - Lisboa, Portugal.

**Keywords:** Pain, Critical illness, Critical care, Behavioral Pain Scale, Critical Care Pain Observation Tool, Pain measurement

## Abstract

**Objective:**

To describe the appropriateness of two behavioral scales, the Behavioral Pain Scale and the Critical Care Pain Observation Tool, for pain assessment in orotracheally intubated patients admitted to intensive care units.

**Method:**

Using the methodology recommended by the Cochrane Center, a systematic literature review was performed in the electronic database EBSCO Host (CINAHL Complete; MEDLINE® Complete; Nursing & Allied Health Collection: Comprehensive; Cochrane Central Register of Controlled Trials; Cochrane Database of Systematic Reviews; Cochrane Methodology Register; Library, Information Science & Technology Abstracts; MedicLatina). Two searches were conducted using the following English terms in the search field: “behavioral pain scale” AND “critical care pain observation tool” AND “behavioral pain scale” OR “critical care pain observation tool”. Two independent reviewers performed the critical evaluation and data extraction and synthesis.

**Results:**

Fifteen studies were included that showed that the Behavioral Pain Scale and the Critical Care Pain Observation Tool are valid and reliable scales for pain assessment in orotracheally intubated patients admitted to the intensive care unit. The scales showed similar psychometric properties and good reliability.

**Conclusion:**

Both scales are adequate for assessing pain in orotracheally intubated patients admitted to intensive care units; however, they exhibit limitations in specific populations, such as trauma, burn and neurosurgical patients. Further studies on the subject and in specific populations are suggested.

## INTRODUCTION

Pain is a subjective symptom that is difficult for health care professionals to evaluate and characterize. Thus, it is important to respect patients' own assessments when they are able to communicate or, alternatively, a properly qualified health care professional's assessment of noncommunicating patients who are intubated, undergoing invasive mechanical ventilation (IMV) and often under sedation.^(1-3)^

Critically ill patients admitted to the intensive care unit (ICU) are subjected to numerous painful procedures, and approximately 75% report severe pain, 30% report pain at rest, and 50% report pain during nursing procedures.^(4)^ Due to the difficulty of assessing and controlling pain, it is often neglected,^(2)^ compromising patient recovery and well-being.^(3)^ The accurate assessment of pain contributes to effective care management; improved adequacy of therapeutic measures, including analgesic and sedative use; and shorter IMV durations and lengths of stay in the ICU.^(3,5)^

Pain control is a patient right and a duty of health care professionals, and the negation and devaluation of pain are ethical errors and failures of excellence in professional practice.^(1,2)^

Thus, when patients cannot self-report pain, health care professionals must resort to the use of validated pain assessment scales,^(3,5)^ such as the Behavioral Pain Scale (BPS),^(6)^ which assesses indicators such as facial expression, upper limb movements and compliance with ventilation (Table 1), and the Critical Care Pain Observation Tool (CPOT),^(5)^ which assesses indicators such as facial expression, body movements, muscle tension and compliance with ventilation in intubated patients or vocalization in extubated patients (Table 2). These two observational and behavioral scales are indicated for the assessment of pain in critically ill patients who are sedated and/or unconscious and undergoing IMV and/or have difficulty with self-reporting pain.^(7,8)^

**Table 1:** Behavioral Pain Scale

Indicator	Item	Score
Facial expression	Relaxed	1
	Partially tightened = brow lowering	2
	Fully tightened = eyelid closing	3
	Grimacing	4
Upper limb	No movement	1
	Partially bent	2
	Fully bent with finger flexion	3
	Permanently retracted	4
Compliance with ventilation	Tolerating movement	1
	Coughing but tolerating ventilation most of the time	2
	Fighting ventilator	3
	Unable to control ventilation	4
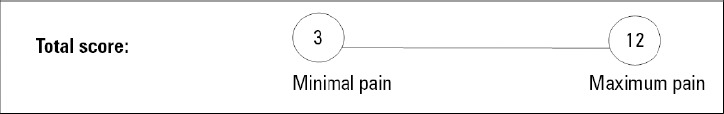		

**Table 2:** Critical Care Pain Observation Tool

Indicator	Item	Score
Facial expression	Relaxed	0
	Tense	1
	Grimacing	2
Body movements	Absence of movements	0
	Protection	1
	Restlessness	2
Muscle tension	Relaxed	0
	Tense or rigid	1
	Very tense or rigid	2
Compliance with the ventilator (intubated patients)/vocalization (extubated patients)	Tolerating ventilator or movement/talking in a normal tone or no sound	0
	Coughing but tolerating ventilator/sighing, moaning	1
	Fighting ventilator/crying out, sobbing	2
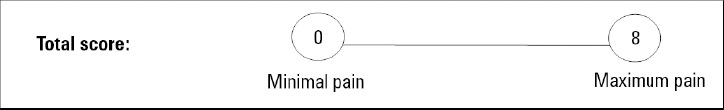		

This is the context of the present systematic literature review (SLR), which aims to identify the appropriateness of the BPS and the CPOT for assessing pain in noncommunicating patients admitted to the ICU.

## METHODS

This SLR followed the methodology recommended by the Cochrane Center^(9)^ and was guided by the following research question: How appropriate are two behavioral scales, BPS and CPOT, for assessing pain in orotracheally intubated patients admitted to the ICU?

Selection criteria were defined and applied according to the PICo method: Participants (adult inpatients older than 18 years), Point of Interest (pain assessment scales: the BPS and the CPOT) and Context (the ICU).

The following exclusion criteria were established: studies conducted with children under 18 years, those including adult inpatients in non-ICU settings, those that used other scales/other strategies to assess pain, qualitative studies, and studies that were not original.

Thus, studies that directly compared the two scales or that mentioned the advantages of using each scale individually were included.

### Search strategy

The Preferred Reporting Items for Systematic Reviews and Meta-Analyses (PRISMA) checklist^(10)^ was used in this SLR as a guide to meet the accepted standards for systematic reviews.

The search was conducted in the EBSCO Host database (Cumulative Index to Nursing and Allied Health Literature (CINAHL) Complete; MEDLINE^®^ Complete; Nursing & Allied Health Collection: Comprehensive; Cochrane Central Register of Controlled Trials; Cochrane Database of Systematic Reviews; Cochrane Methodology Register; Library, Information Science & Technology Abstracts; MedicLatina). A search was also manually conducted in the references of published studies on the subject.

Two independent searches of the databases were performed. For both searches, the search terms "behavioral pain scale" and "critical care pain observation tool" were introduced. For the first search, the Boolean operator "AND" was used; for the second, the Boolean operator "OR" was used. The review was limited to studies published in Portuguese or English, and both searches were conducted without a time limit.

The titles were read first, followed by the abstracts and then the full text of the articles found in the search in order to select those that answered the research question.

The studies were read and their methodological quality (MQ) was evaluated by two independent researchers to ensure critical evaluation during article selection. In cases of disagreements between the researchers, a third evaluator was asked to review the article.

The MQ of the studies was assessed using instruments from the Joanna Briggs Institute (MAStARI).^(11,12)^

Before the investigators conducted their evaluations, it was established that only studies with high MQ, i.e., those with a score of 8 to 10 on the MAStARI Checklist for Diagnostic Test Accuracy Studies,^(12)^ would be included (Table 3).

**Table 3:** Characteristics of the selected studies

Author/country	Objectives	Methods/MQ^(12)^	Participants	Results
Gélinas et al.,^(5)^ Canada	Validate the CPOT during painful and nonpainful procedures	Quantitative observational study The CPOT was administered at 3 timepoints: at rest, during a painful procedure and 20 minutes after the procedure (9 assessments) MQ = 9	Convenience sample of 105 critically ill adult patients admitted to the ICU for cardiac surgery	The CPOT showed good interrater reliability (ICC = 0.52-0.88), adequate content validity (0.88 - 1.0) and criterion validity (patients who reported pain: 1.62 - 3.65) The results show the need to validate the CPOT in different critically ill patient populations
Morete et al.,^(7)^ Brazil	Translate and culturally adapt the BPS to Brazilian Portuguese and perform validation	Methodological study with quantitative analysis The cultural adaptation of the BPS to Brazil and the study of its psychometric properties were performed MQ = 10	Convenience sample of 100 adult patients admitted to the ICU, undergoing IMV and with or without sedation and analgesia	The BPS showed easy application and reproducibility, with adequate agreement between the two evaluators (ICC = 0.807, 95% CI = 0.727 - 0.866) and with adequate internal consistency (α= 0.501), and its adaptation to Brazil was satisfactory
Nürnberg et al.,^(13)^ Sweden	Validate the Swedish version of the CPOT during painful and nonpainful procedures	Observational descriptive study 240 independent observations were performed by two team members before, during and 15 minutes after painful and nonpainful procedures MQ = 10	Convenience sample of 40 conscious and unconscious intubated adult patients admitted to the ICU	The validation of the CPOT showed good interrater reliability (ICC = 0.84), internal consistency evaluated during the assessments (between: α = 0.31 - 0.81) and adequate discriminant validity
Rijkenberg et al.,^(14)^ the Netherlands	Compare the discriminant validity and reliability of the CPOT and BPS simultaneously in patients under IMV admitted to an adult ICU	Observational prospective study Assessment of pain in patients undergoing IMV using the BPS and CPOT at rest, shortly before and during painful and nonpainful procedures MQ = 10	Convenience sample of 68 patients admitted to the ICU under IMV The sample was divided into three subgroups according to RASS scores	Both scales (BPS and CPOT) were reliable and valid for pain assessment in the ICU There was good interrater reliability (ICC = 0.75 for the CPOT and ICC = 0.75 for the BPS); good internal consistency (α = 0.71 for the CPOT and 0.70 for the BPS) Although most of the indicators in both scales increased with a painful procedure, only those in the BPS increased in association with a nonpainful procedure
Liu et al.,^(15)^ China	Evaluate and compare the reliability and validity of the BPS and the CPOT for pain assessment in intubated and nonintubated critically ill patients	Observational prospective study A total of 608 pain assessments were performed using the CPOT and BPS (BPS and BPS-NI) before and during painful and nonpainful procedures MQ = 9	Convenience sample of 117 critically ill adult patients admitted to the ICU	The BPS and CPOT were found to be reliable and valid to assess pain in intubated and nonintubated patients The results showed good interrater reliability (ICC = 0.973 for the CPOT and ICC = 0.955 for the BPS); good internal consistency (α = 0.795 for the CPOT and 0.791 for the BPS) and reliability of 0.950 (CPOT) and 0.941 (BS)
Al Darwish et al.,^(16)^ Saudi Arabia	Determine the reliability and validity of nonverbal pain assessment tools in critically ill patients (BPS, NVPS and CPOT)	Descriptive observational study with quantitative analysis Three pain assessment instruments – the BPS, CPOT, NVPS – were administered before, during and after painful and nonpainful procedures for a total of 240 evaluations MQ = 9	Convenience sample of 47 critically ill noncommunicating patients undergoing IMV admitted to the ICU	The BPS was the most valid and appropriate instrument to assess pain in noncommunicating patients admitted to the ICU due to the characteristics of its subscales; however, the CPOT was considered an appropriate alternative. The results showed good interrater reliability (ICC = 0.80) and good internal consistency (α = 0.95 (CPOT); 0.95 (BPS); 0.86 (NVPS)) The NVPS was not sensitive to assess pain in these patients
Rahu et al.,^(17)^ United States	Identify the most appropriate scale for pain assessment in intubated patients Determine the validity and sensitivity of six pain scales	Descriptive study Observations were made by two independent investigators in communicating and noncommunicating intubated patients before and during nonpainful and painful procedures using six scales (NVPS; BPS; Comfort; Faces; Face, Legs, Activity, Cry, Consolability; NRP) MQ = 9	Convenience sample of 50 communicating patients and 100 patients who were unable to communicate verbally who were intubated and undergoing IMV	All pain scales had moderate to high correlation with the self-report of patients during endotracheal suction (painful procedure) All scales were sensitive in obtaining the patient's pain response in all phases (p < 0.001) Both the patients and the investigators assessed the highest pain on the Faces scale, which reveals that some caution in its use is necessary
Chanques et al.,^(18)^ United States	Compare the psychometric properties of three pain assessment scales (the BPS/BPS-NI, CPOT and NVPS) in intubated and nonintubated patients unable to self-report pain	A total of 258 assessments of pain, sedation (RASS) and delirium (CAM-ICU) were performed by at least one investigator and one nurse in 30 patients before, during and 10 minutes after routine procedures MQ = 10	Convenience sample of 30 adult patients; RASS > -4 in patients who were unable to self-report their pain intensity	The three scales showed good psychometric properties in the assessment of pain in intubated and nonintubated patients unable to self-report their pain intensity The BPS and CPOT showed better reliability (κ = 0.81 for both) and internal consistency (α = 0.80 - BPS; α = 0.81 - CPOT). The BPS was classified as the most feasible scale, with the highest score on the category of "easiest to remember"
Bourbonnais et al.,^(19)^ Canada	Determine the appropriateness of the CPOT as an instrument for assessing pain in adult patients under IMV admitted to the ICU	Descriptive study Applied a data collection instrument (CPOT, record of sedation, analgesia and performed interventions, and open questions to nurses related to the use of CPOT) MQ = 9	Convenience sample of 23 nurses who used the CPOT to assess 23 patients	Each patient was assessed five times, for a total of 115 evaluations 75 assessments indicated that the patient presented pain (mean = 3.03) Pain assessment and identification of pain episodes occurred more frequently when the CPOT was applied Nurses stated that the scale was easy to use and that it would be useful to apply it in practice for the identification of pain in patients undergoing IMV admitted to the ICU
Vadelka et al.,^(20)^ Italy	Analyze the degree of compatibility between the CPOT and the BPS/BPS-NI when evaluating pain	Cross-sectional observational study A total of 528 pain assessments were performed on patients admitted to the ICU before and after two procedures (one painful and one nonpainful) MQ = 9	Convenience sample of 33 patients admitted to the ICU	Both tools were considered valid and reliable, capable of detecting the intensity of pain in critically ill patients even under high levels of pharmacological sedation There were no significant differences (p > 0.05) between patients with different levels of sedation or analgesia
Severgnini et al.,^(21)^ Italy	Compare the CPOT and BPS for the assessment of pain in conscious and unconscious patients	Observational study A total of 303 consecutive observations were performed over 3 days after admission to the ICU Measurements with both scales were obtained 1 minute before, during and 20 minutes after nursing procedures The VAS score was recorded, whenever possible, only in conscious patients MQ = 10	Convenience sample of 101 patients (conscious: n = 41; unconscious: n = 60)	Both the BPS and CPOT can be used to assess pain intensity in critically ill conscious and unconscious patients undergoing mechanical ventilation but have different sensitivities and specificities Comparison of the CPOT and BPS at three different times using Cohen's kappa (before k = 0.69, during = 0.64 and after = 0.66) showed good correlation (k > 0.6) This study suggests that the CPOT is equivalent to the BPS because no scale has better sensitivity and specificity The criterion validity between the VAS and BPS (rs = 0.56; p < 0.0001) and the VAS and CPOT (rs = 0.48; p <0.0001) showed a strong correlation
Hylén et al.,^(22)^ Sweden	Translate and validate the BPS for critically ill patients	Observational descriptive study with quantitative analysis The scale was applied before and after procedures considered potentially painful (repositioning) MQ = 9	Convenience sample of 20 critically ill patients admitted to the ICU (10 intubated and 10 nonintubated patients)	The Swedish version of the BPS is adequate for pain assessment in patients unable to self-report pain The discriminant validity for the assessments before, during and after the procedure obtained a percentage agreement of 28%, with 95% CI (relative position of -0.08 to +0.02; relative concentration of -0.06 to +0.08; relative variance of classification of 0.000 - 0.002) and a reliability of 85%.
Frandsen et al.,^(23)^ Denmark	Validate the Danish version of the CPOT for patients admitted to the ICU without a sedation protocol	Quantitative, descriptive, observational study Patients were observed before, during and 15 minutes after nonpainful and a painful procedures (6 observations performed by two independent observers). MQ = 10	Convenience sample of 70 critically ill patients admitted to the ICU and undergoing mechanical ventilation without sedation	CPOT shows good reliability and interrater agreement (ICC > 0.90), internal consistency (α> 0.70) and significant correlation between the CPOT values and the reported pain (p < 0.05)
Linde et al.,^(24)^ Iceland	Validate the CPOT for pain assessment during painful and nonpainful procedures	Observational descriptive study Observational data were collected during painful procedure and a nonpainful procedure MQ = 9	Convenience sample of 30 intubated patients after cardiac surgery	The results support the viability and reliability of the CPOT in the assessment of pain in adult patients The mean CPOT scores showed a significant increase only during the painful procedure (+3.04; 95% CI 2.11 - 3.98; p < 0.001)
Topolovec-Vranic et al.,^(25)^ Canada	Evaluate the validity and clinical utility of the NVPS-R and the CPOT in a trauma and neurosurgical patient population	Prospective descriptive study Assessment of pain using the NVPS-R and CPOT at three separate times: before, during and after a painful and a nonpainful procedure MQ = 9	23 nurses (12 assessed patient pain using the CPOT and 11 using the NVPS-R) Convenience sample of 66 patients admitted to the adult ICU (34 communicating and 32 noncommunicating patients)	The CPOT has a greater validity than the NVPS-R for pain assessment in critically ill noncommunicating patients, particularly those with neurological and trauma injuries The interrater reliability was higher for the CPOT (0.60 - 0.97) than for the NVPS-R (0.34 - 0.92). The self-reported pain and assessment performed by nurses showed a moderate correlation in both scales (NVPS-R: σ = 0.313 and p < 0.001; CPOT: σ = 0.435 and p < 0.001).

CPOT - Critical Care Pain Observation Tool; MQ - methodological quality; ICU - intensive care unit; ICC - intraclass correlation coefficient; BPS - Behavioral Pain Scale; IMV - invasive mechanical ventilation; 95% CI - 95% confidence interval; RASS - Richmond Agitation-Sedation Scale; BPS-NI - Behavioral Pain Scale-Non-Intubated; NVPS - Nonverbal Pain Scale; CAM-ICU - Confusion Assessment Method in the Intensive Care Unit; VAS - Visual Analogue Scale; NVPS-R: Nonverbal Pain Scale-Revised.

## RESULTS

The first search with the Boolean operator AND resulted in 186 studies; 32 of them were excluded due to duplication, 109 after the title was read and 36 after the abstract was read. Of the 36 that were excluded after the abstract was read, 11 were excluded because the full text was not available, 3 because they were not in English or Portuguese, 2 because they were duplicates, 5 because they used other pain assessment scales, 12 because they were not original, 1 because it focused on pain control interventions, 1 because it focused on drug administration and 1 because it focused on a specific population that did not meet the inclusion criteria of the present review).

**Figure 1:**
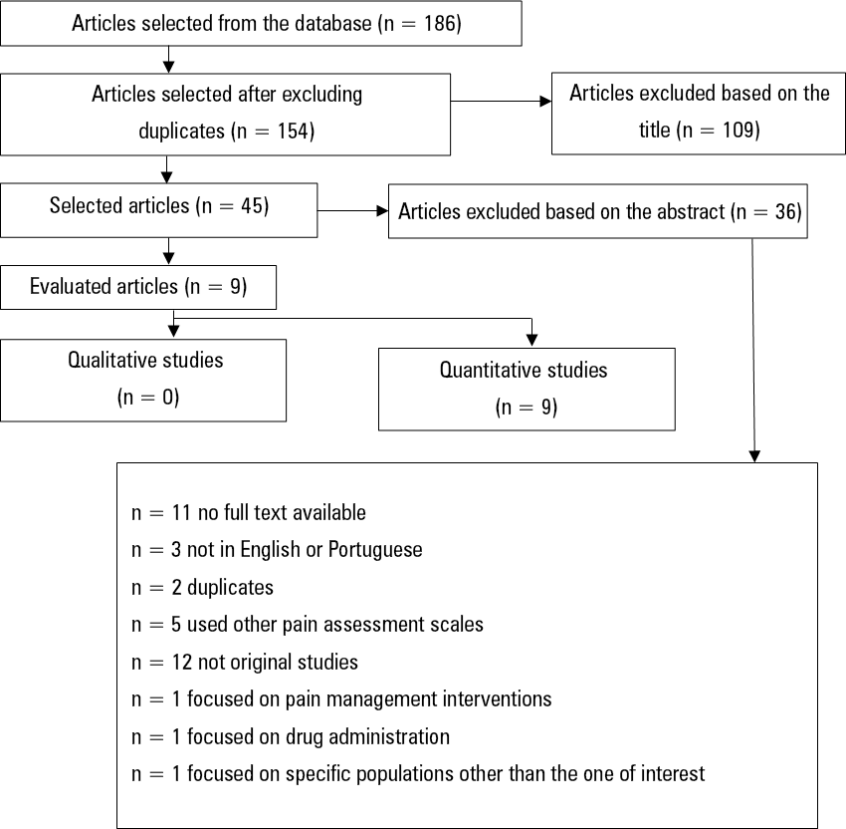
Flow chart of the included studies - first search.

After reading the abstract, 9 studies were selected, and these studies remained in the sample after the full text was read (Figure 1). The 9 studies showed high MQ according to the cited criteria (Table 3).

The 9 selected studies were conducted in different countries: Sweden,^(13)^ the Netherlands,^(14)^ China,^(15)^ Saudi Arabia,^(16)^ the United States,^(17,18)^ Canada,^(19)^ and Italy.^(20,21)^ They were published in 2011,^(13)^ 2014,^(18)^ 2015,^(14,15,17)^ 2016^(16,19,21)^ and 2017.^(20)^

The second search with the Boolean operator "OR" resulted in 853 studies; 208 of them were excluded due to duplication, 601 after the title was read, 22 after the abstract was read and 12 after they were read in full. In the various elimination stages, studies were excluded because they were not in English or Portuguese, because they focused on a specific population other than the one considered in this review (children or nonintubated patients, for example), because they were duplicates, because they were not original studies, or because they were studies on other topics (validations of other scales, for example).

**Figure 2:**
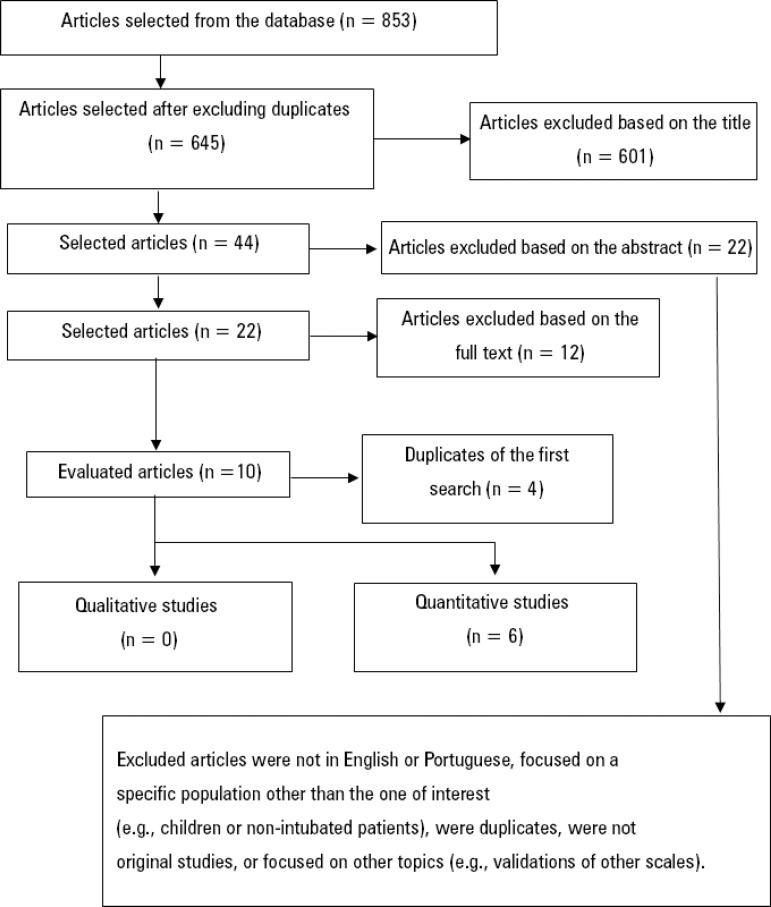
Flow chart of the included studies - second search.

After the full text was read, ten studies were selected, four of which were eliminated because they had already been included in the first search with the Boolean operator AND; thus, six studies were included in the sample (Figure 2), and all of them had high MQ scores^(12)^ (Table 3).

The six selected studies were conducted in different countries: Brazil,^(7)^ Sweden,^(22)^ Denmark,^(23)^ Iceland^(24)^ and Canada.^(5,25)^ They were published in 2006,^(5)^ 2013,^(24,25)^ 2014^(7)^ and 2016.^(22,23)^

After the results of the two searches were compared, 15 studies were included in this SLR. All of the studies were quantitative, and their sample sizes ranged from 20^(22)^ to 150^(17)^ patients.

The BPS was developed and tested in 2001 by Payen et al.^(26)^ on a sample of 30 patients with medical and surgical diagnoses undergoing IMV (269 observations), and it showed good validity and reliability in the study population.

During the validation process, the Brazilian version^(7)^ had the lowest internal consistency (Cronbach's alpha = 0.50); the remaining versions had internal consistency ranging between α = 0.79^(15)^ and α = 0.95.^(16)^ All versions showed good interrater agreement (intraclass correlation coefficient - ICC = 0.80;^(7,16)^ 0.97^(15)^) and good criterion validity.

There were differences in the correlations between assessments performed before, during and after a painful procedure,^(15,22,26)^ and the discriminant validity for assessments performed before, during and after the procedure obtained a percentage agreement of 28%. However, in one of the studies, the discriminant validity was less supported because it increased during exposure to a nonpainful stimulus.^(14)^ Whereas in the original validation study, there were negative correlations between the pain score and the administered midazolam and fentanyl doses,^(26)^ in the validation study for Saudi Arabia, routine procedures, such as secretion aspiration, caused pain in all patients regardless of the administration of analgesia.^(16)^

The CPOT was developed in 2006 in French, in Quebéc, Canada, by Gélinas et al.^(5)^ and was validated on a convenience sample of 105 intubated cardiac surgery patients (33 unconscious and 99 conscious) before and after extubation. The CPOT showed good interrater reliability (ICC: 0.52 - 0.88), adequate content validity (0.88 - 1.0) and criterion validity (patients who reported pain (1.62 - 3.65)), and discriminant validity was evidenced by higher CPOT scores during painful procedures than at rest (t = -9.01 - -15.96; p < 0.001). Spearman correlations of 0.40 - 0.59 (p < 0.001) showed that the patients' self-reported pain intensity scores were moderately correlated with the CPOT scores.^(5)^

The review found that the CPOT had been validated/revalidated with communicating and noncommunicating patients admitted to the ICU. It was validated for the Swedish population on a sample of 40 intubated patients,^(13)^ for Iceland on a sample of 30 patients intubated after cardiac surgery,^(24)^ for Canada on a sample of 23 intubated patients,^(19)^ for the United States on a sample of 30 intubated patients,^(18)^ in Toronto on 66 trauma and neurosurgical patients (34 communicating and 32 noncommunicating),^(25)^ for the Netherlands on 68 patients undergoing mechanical ventilation,^(14)^ for Italy on a sample of 101 patients (41 conscious and 60 unconscious),^(21)^ for China on a sample of 117 critically ill ventilated patients,^(15)^ for Denmark on 70 patients undergoing IMV without sedation,^(23)^ and for Saudi Arabia on 47 noncommunicating critically ill patients.^(16)^

Validation of the CPOT revealed good reliability (ICC of 0.75^(14)^ - 0.95^(15)^) and internal consistency (Cronbach' alpha of 0.70^(23)^ - 0.973^(15)^) and good criterion validity. There was a significant correlation between the pain intensity scores reported by communicating patients and the CPOT scores;^(5)^ the scores increased significantly when patients were exposed to painful procedures rather than nonpainful procedures, indicating criterion validity.^(13-16,20,23-25)^ Significant correlations were also found between CPOT scores and mean arterial pressure (p = 0.32 - 0.45).^(25)^ and general vital signs before, during and after a painful procedure;^(20)^ however, further studies should be conducted to explore the role of vital signs in pain.^(25)^

Table 3 shows the objectives, methods and assessment of MQ,^(12)^ the participants and the results of the selected studies.

## DISCUSSION

The detection, quantification and treatment of pain in critically patients have long been a concern of health care professionals. Nevertheless, pain is common in critically ill patients, regardless of their clinical condition, and its accurate assessment using appropriate instruments allows for the use of more beneficial therapeutic measures.

The BPS and CPOT have good psychometric properties and good reliability in intubated and nonintubated patients in the ICU who are unable to self-report their pain,^(8,14-16,18,20,21,23,24)^ and both scales should be used for pain assessment in this patient population.^(18,21)^

The BPS is considered applicable to critically ill patients who are sedated, unconscious or have difficulty self-reporting pain, especially those undergoing IMV, given that one of its three domains pertains specifically to compliance with ventilation. In turn, the CPOT, in addition to the domain intended for patients undergoing mechanical ventilation, has a vocalization domain; thus, it can also be used with extubated patients, unlike the BPS, which is only intended for use with patients undergoing IMV.^(8)^

Some authors have stated that the BPS is the more viable,^(18)^ specific,^(21)^ reliable, valid^(16)^ and sensitive tool for assessing patient pain; however, the CPOT is a good alternative.^(16,21)^ Other authors consider the CPOT the scale of choice because its discriminant validity is more supported because the score does not increase with a nonpainful stimulus, contrary to what has been observed with the BPS.^(14)^

In addition, there was an increase in the pain assessment scores of both the BPS and the CPOT when a painful procedure was evaluated.^(13-17,20,23-25)^ Both instruments are sensitive to painful procedures, with an observed increase in the various indicators that constitute both scales.^(14-17)^ The main parameters with major changes are facial expression in the BPS^(18,21)^ and muscle tension/stiffness, facial tension and ventilator tolerance/cough in the CPOT.^(19,21)^

During painful procedures, there is a significant correlation between pain scores and vital signs, specifically blood pressure;, i.e., the higher the pain score, the higher the blood pressure will be.^(13,20)^ Although this correlation has been observed, some authors suggest that further studies be conducted to explore the relationship between vital signs and pain.^(25)^

Although some consider the CPOT to have good validity for assessing pain in noncommunicating critically ill patients, particularly those with neurological and traumatic injuries, and in neurosurgical patients,^(25)^ others argue that its application to patients with brain injuries,^(27,28)^ cognitive deficits or burns^(28,29)^ is a limitation.

There is still no consensus regarding levels of consciousness, sedation and analgesia because some authors found no significant differences in the application of the two scales to patients with different levels of sedation or analgesia;^(20)^ however, other authors found significant differences during and after nursing care in conscious and unconscious patients.^(21,28)^

In their practical application, both the BPS and CPOT are considered by health care professionals to be useful tools in the ICU setting because they are easy to use and remember.^(7,18-20,25)^ Their application contributes to an increased frequency of assessments and, consequently, to the reduced administration of analgesics and sedatives.^(3,30)^ Researchers have suggested using the two scales simultaneously, as doing so may result in more accurate pain detection and assessment.^(21)^

Although the use of the BPS and CPOT scales has been shown to have positive effects on pain management in patients admitted to the ICU, experimental studies are recommended.^(28)^

## CONCLUSION

This systematic literature review found several studies validating the Behavioral Pain Scale and the Critical-Care Pain Observation Tool for use with orotracheally intubated critically ill patients from various cultures, and both instruments were found to be valid and reliable for assessing pain in intubated patients admitted to intensive care units.

Both instruments were sensitive when applied during painful procedures, showing increases in various indicators: facial expression on the Behavioral Pain Scale and muscle tension/stiffness, facial tension and ventilator tolerance/cough on the Critical-Care Pain Observation Tool, and blood pressure on both scales.

There is, however, no agreement regarding the administration of the scales in patients with different levels of consciousness, sedation and analgesia. However, it appears that the use of at least one of the scales helps to increase the frequency of assessments and, consequently, reduces the administration of analgesics and sedatives. In this regard, it is essential that health care professionals use at least one of the two analyzed scales for pain assessment in intubated patients, with the goal of improving the care provided.

Further studies with an experimental design covering different critically ill patients populations admitted to intensive care units, namely, trauma, burn and neurosurgical patients, are suggested.
